# A Novel Human Autonomy Assessment System

**DOI:** 10.3390/s120607828

**Published:** 2012-06-08

**Authors:** Marco Munstermann, Torsten Stevens, Wolfram Luther

**Affiliations:** 1 Health & Care Solutions (HCS), Fraunhofer IMS, Finkenstr. 61, 47057 Duisburg, Germany; E-Mail: torsten.stevens@ims.fraunhofer.de; 2 Computer Science and Applied Cognitive Science, University of Duisburg-Essen, Lotharstr. 63, 47057 Duisburg, Germany; E-Mail: luther@inf.uni-due.de

**Keywords:** activities of daily living, activity recognition, concur task trees, context awareness, data mining, Petri nets, probabilistic models, task models, transition system miner

## Abstract

This article presents a novel human autonomy assessment system for generating context and discovering the behaviors of older people who use ambulant services. Our goal is to assist caregivers in assessing possibly abnormal health conditions in their clients concerning their level of autonomy, thus enabling caregivers to take countermeasures as soon as possible.

## 1. Introduction

The number of people in Europe age 65 and over will rise from 75 million in 2004 to 133 million in 2050 [1]. Even if many older people try to stay active, it is unavoidable that their physical abilities will decline. Moreover, their cognitive skills will also undergo a negative change over time. This can begin with trivial incidents like forgetting where they have placed their glasses and possibly end in an abnormal state (e.g., dementia or Alzheimer's disease [AD]).

When misplacement of objects turns into a more serious problem that affects daily living (e.g., managing their medication [2]), people are often no longer able to live independently. To most people, this is a frightening prospect. That is where the idea of Ambient Assisted Living (AAL) comes in. By utilizing ambient technology, people (mostly elderly) are able to live in their own homes for as long as possible.

Fortunately, the objection to institutional care coincides with the (future) financial resources of the health care system. In Germany roughly 69% (as of 2009) of all patients receiving professional care can be treated in their own homes. The remaining 31% are either partly—through day care—or completely institutionalized. Either way, the reason they are receiving professional care is that an independent authority states they are considerably limited in the activities of daily living (ADLs).

Despite the fact that a great deal of work has been done on activity recognition [3] and anomaly detection [4], as far as we know, only one existing study (*i.e.*, [5]) uses *a priori* (graphical) model-based knowledge to define the set of activities the subsequent anomalous behavior detection will rely upon. While Hong *et al.* utilize an ontology-based approach, we define ADLs using task models. Their main intention is to cope with the uncertainty of sensor data. Our choice of task models as a definition language was driven by the concept of involving caregivers during the system design phase. This approach will strictly distinguish between impersonalized knowledge during context generation and personalized knowledge for anomalous behavior discovery.

Changes in behavior concerning ADLs can be seen as an early indicator of autonomy loss [6]. One of the earliest assessments of human behavior related to the ability to live independently was conducted by Katz *et al.* [7]. A significant drawback of questionnaires such as those used in that study is that elderly people tend to lie about their difficulties (due either to fear of the consequences or to shame).

Later, Lawton [8] constructed a hierarchical taxonomy of behavioral competence. He presents five major categories, from simple to complex: health, functional health, cognition, time use and social behavior. The hierarchy demonstrates that the complex tasks rely heavily on the simple ones. He states that residential behavior is closely related to cognitive competence.

Telemonitoring products concentrate on well-being at the *health* [8] level (e.g., 24-hour ECG monitoring). We tackle the automatic detection of behavioral competence at *the, functional health* level. Basically, we focus on the physical (sometimes called basic) and instrumental aspects of ADLs. So far, we have defined the following ADLs: going to the toilet, transferring, waking/sleeping, dressing, eating, washing, bathing, combing and napping.

To a certain degree, people reveal their current context (activity, location, identity and time) [9] by interacting with their environment (e.g., by opening the doors of a cupboard). Assuming additional semantic information is provided, it is possible to draw inferences about any activity currently being performed simply by augmenting their surroundings with the appropriate sensors.

When considering an individual's daily habits, some repetitive patterns can easily be observed, starting with the person's wake-up routine [10]. After collecting enough context information about a specific person to create a temporal relationship model of his/her daily activities, we can assess to what extent the current day's pattern of activities matches those of previously observed days.

The system described in this article is intended as an aid to the caregiver. Therefore, one highly influential concern in its design is the awareness that in practice the caregiver will have very limited time to interact with an assessment tool. To this end, we have created a novel human autonomy assessment system (HAAS) at different levels: during the specification phase, a clear and easy-to-understand language had to be chosen so the developer can profit from the domain knowledge of the caregiver. When it comes to visualization, we had to find a highly condensed behavioral information representation of the client's daily living skills.

We use the term *client* to refer to the person the caregiver looks after. The word *patient* is typically used by medical staff. However, *patient* has the disadvantage that the person might feel as if he/she were in a hospital rather than in his/her own home. Since caregivers usually interpret themselves as service providers, they tend to call their customers *clients*. Of course, questioning the person directly usually leads to a simple and nearly obvious answer: They prefer the word *occupant*. Nevertheless, in its current development state the system is primarily meant to assist caregivers; therefore, we will use the term *client* throughout this article.

Let us assume the caregiver arrives at the home of one of his clients. He has not seen that person for almost 24 hours and does not know what has actually happened in that time. Ideally, the client will report on any problems he has faced during the caregiver's absence. However, sometimes—as with the questionnaires—clients might lie or simply neglect to inform the caregiver about relevant issues.

This is where HAAS can help the caregiver. The metric value HAAS will generate can give an indication in terms of how normal the previous day was. When the caregiver perceives a low metric value, he/she will automatically know that something was unusual. In Section 6 we show that the metric values are quite relevant.

By comparing the previous day to the typical behavior of that specific client, the caregiver can also find out when and where things diverged from the norm. HAAS provides a simple interface to highlight those spots. The caregiver can then use this information to discuss the findings with his client.

Currently, the system is based on the information pull principle. This means that the caregiver has to actively seek advice from the system (on a daily basis). Theoretically, we could instigate a system whereby the caregiver would receive a warning whenever a client deviates from his/her typical behavior. However, simply informing the caregiver about such an occurrence is not sufficient. It would only make sense if someone could check on the client outside of the regular schedule. Whereas that is typically the day-to-day business of medical staff, ambulant care services usually do not have enough human resources to do so. Since it is not possible to respond to such a warning, we decided not to develop an alarm system.

The remainder of this article is structured as follows. It begins with an overview of related work. Next, the concept is presented in its entirety and then subdivided into its components, which are discussed in greater detail. Finally, we describe the metric used to rate human behavior and some initial steps in evaluating the system.

## 2. Related Work

Since caregivers have indicated that clients are not very accepting of the notion of intrusive sensors affecting their privacy, we will mainly focus this overview on non-intrusive sensors (*i.e.*, systems not using microphones or cameras). Hong *et al.* [5] and Kart *et al.* [11] had quite similar findings. While [5] describes a framework based on anonymous binary sensors for monitoring ADLs which focuses on reasoning under uncertainty, [11] investigates the use of electronic technology to assist caring family members by means of a pilot project.

Bao and Intille [12] have demonstrated the application of multiple accelerometers attached to the body Using decision tree classifiers, they have successfully recognized one out of twenty activities (e.g., walking, sitting, standing or brushing teeth) with a probability of more than 80%. For training purposes, the data was labeled in advance by the person under observation.

One advantage of wearable sensors is that they can detect even outdoor activities. On the other hand, the wearer has to ensure their power supply (*i.e.*, recharging or replacing of batteries) and cope with the sometimes cumbersome way of attaching/wearing them. Having older people as a target audience makes it unlikely that they will always wear and charge the sensors (correctly). It is not improbable that they will simply forget to put them on. Although accelerometers do not affect one's privacy, they do impair one's personal comfort.

Another type of sensing is presented by Wyatt *et al.* [13]. Instead of using accelerometer data, they draw their inferences from pervasive light-weight sensor data. Therefore, every meaningful (to the intended application) object in the household is equipped with a radio-frequency identification (RFID) tag. The user must wear the counterpart—a mobile RFID reader (usually near the wrist of his/her dominant hand). As soon as the person grabs a tagged object, its identity is recognized by the system. Finally, inference is made on the basis of common sense gathered from a knowledge base (e.g., the World Wide Web). A web-mining technique is applied to search for activities related to the object being manipulated. Let us assume the person has just reached for his/her toothbrush. There might be a web site explaining the correct procedure for brushing one's teeth. The co-occurrence of the words *toothbrush*—the object—and *brushing teeth*—the corresponding activity—will raise the probability that the person is currently brushing his/her teeth.

From an academic point of view, this type of unsupervised learning is very interesting. In a practical scenario, every object belonging to an activity to be recognized would have to be tagged. Moreover, the system relies on the client to wear his/her RFID reader.

Wilson [14] has described an activity recognition (and tracking) system that uses mainly binary sensors. Instead of equipping the person or objects with tags, he places simple sensors in the environment. Usually these types of binary sensors are installed in home security systems. His approach is based on a Rao-Blackwellised particle filter offering a sample-based approximation of probability densities. The system not only recognizes activities; it can also track movements and even identify people (using RFID readers placed only at entrances and exits). Currently, it is the person who carries the RFID tag and the readers are fixed. There is no need for the user to be concerned with charging the reader, nor must he/she attach any sensors to his/her body. In terms of ease of use, this system definitely outperforms the former two solutions in real application.

Another way to group recent work is to relate it to the test bed (laboratory) it came from. Worldwide, there are many prominent research facilities for ambient assisted technology. Several of them are discussed below.

One exponent of the PlaceLab, which is operated jointly by MIT's House_n and TIAX, LLC, is Munguia Tapia *et al.* [15]. Based on a sensor setup similar to Wilson's [14], researchers at the PlaceLab demonstrate the use of naive Bayes classifiers for activity recognition. Since the approach is based on supervised learning, training data is necessary

Another research site is located at the University of Florida: the Gator Tech Smart House (GTSH). Exemplary work on the GTSH has been carried out by Kim *et al.* [16]. Two approaches using motion sensors are presented: a Hidden Markov Model (HMM) and a Conditional Random Field (CRF).

Washington State University hosts the CASAS Smart Home Project. Rashidi *et al.* [17] describe the methods used there. First, a mining method is employed. Then, the activities discovered by the mining are clustered. Finally, a HMM identifies routines in those activities.

The MavHome is the domestic test bed developed at the University of Texas at Arlington. Cook [18] describes a holistic approach comprising automated discovery, prediction and decision making. The Episode Discovery (ED) algorithm is used to recognize activity patterns. An enhanced version of the LZ78 algorithm—the Active LeZi (ALZ) algorithm—is used to make predictions. For decision making, a hierarchical HMM is employed.

Aztiria *et al.* [19] identifies three different groups of sensor types: context sensors, motion sensors and sensors related to objects. Although this work concentrates especially on the last group of sensors, sensors from the other two groups are also applied.

At Carnegie Mellon University, Cai [20] has worked on an interesting study with an aim similar to ours. The most obvious difference lies in his use of a mobile sensor platform.

The activity inference of the SCAN framework presented in [21] is also based on handling artifacts. In addition to RFID technology for tagging objects, accelerometers and video cameras are employed. The tests took place in the author's lab in Mexico.

Le *et al.* [22] presents a work in progress from the PROSAFE project [23]. This European project seeks to automatically assess the dependency of elders by using only non-invasive sensors. Activity recognition is achieved by HMM.

The ALISA project is another European research project promoting an experimental platform for evaluation of ambient technology dedicated to the elderly. Noury [24] describes the platform in detail.

The results that have been cited so far come from academic labs; very few commercial products exist. Two examples for those few commercial products are the QuietCare system [25] and a product called BeClose [26].

As far as we know, none of the existing systems enable formal definition of which activities are of interest and how they should ideally be performed using a graphical model description. Furthermore, none of the systems is based on the context information generated by formal definitions to detect anomalies in daily behavior. Formalisms are normally used, such as those presented by Benghazi *et al.* [27]. Their main issue is verification in terms of timeliness and safety (*i.e.*, deadlock freeness).

Thus, the system presented is of great practical relevance since self-labeled sensor data is not applicable for senior citizens (especially if they already suffer from dementia) and labeled data is time intensive to gather and therefore very costly.

## 3. System Overview

**Figure 1.**
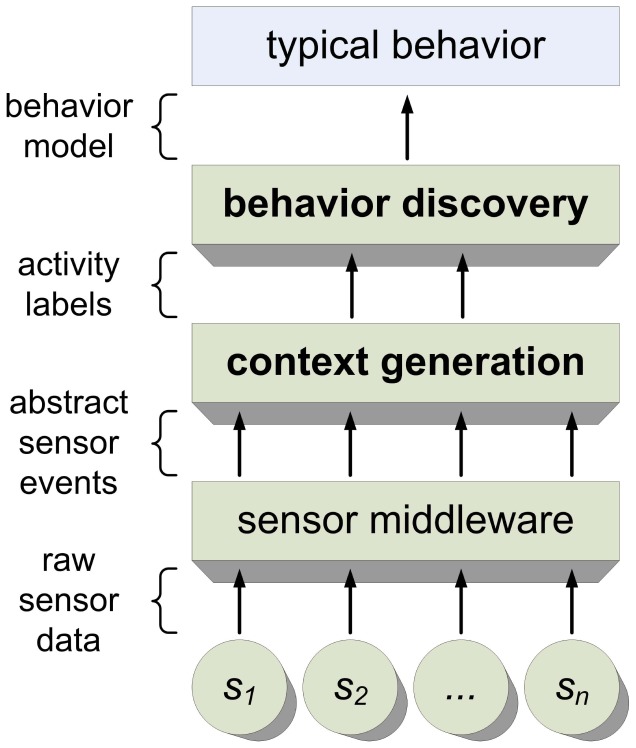
System overview with two main building blocks: context generation module and behavior discovery module.

HAAS consists of two main building blocks: *context generation* and *behavior discovery* (see Figure 1), which will be considered in this section. The third building block, *sensor middleware*, is not as unique as the other two. A good overview of promising middleware platforms can be found on this website [28]. Many AAL systems utilize the OSGi framework (e.g., Coronato [29]). Because it is dealt with thoroughly elsewhere, we will explain sensor middleware only briefly and without particular emphasis.

The *context generation* module will interpret events coming from the sensor middleware and output the corresponding activities performed (ADLs). To define those activities, the system uses a model-driven, top-down approach. By first defining ADLs using models, we can then execute a conformance check in real-time. The models provide a source of common reference (see Section 3.2 for further details). There is no need for supervised and often tedious manual labeling of datasets.

The second main module, *behavior discovery*, will use this contextual information as input. By observing daily habits (related to the activities modeled and enabled using the setup tool), HAAS will construct the typical schedule of activities for a specific person. Because human activities tend to be complex and often vary, the behavior model is probabilistic. Using this typical behavior of more or less probable sequences of activities as a reference, current activity sequences can then be rated in relation to previously observed patterns.

In the current phase of development, the system is primarily intended to be a logging and classifying device for the caregiver. No automatic actions whatsoever are taken by the system.

Sensor middleware and context generation are capable of real-time operation. Each sensor data or sensor event triggers an action within those modules. The behavior discovery works on a daily cycle. After a complete day (*i.e.*, from getting up on one day to getting up the next day) the behavior discovery will produce/update the typical behavior and output a metric value (see Section 4). The reason we have chosen a daily base is rooted in the nature of the human biological rhythm. Like no other, the circadian rhythm has a great influence on our life. Similar to our vital signs, our biological clock also follows this daily cycle (Hofman and Swaab [30]). For example, depending on cultural habits, people usually have three (two or four) meals a day.

Our sensing approach is based on reliable detection of object use, operating primarily with binary sensors. This way a maximum of the user's privacy can be preserved, *i.e.*, especially compared to audio and video surveillance systems. For instance, binary sensors are not capable of detecting the person's identity. Object use is a good indication of the activity the user is currently performing. Admittedly, it comes at the expense of having to install a great many sensors.

Even if the user might change his/her habits or surroundings or rearrange furnishings over time, the objects used to perform ADLs (e.g., using the toilet or the refrigerator) basically remain the same. Manipulating a certain object will effectively distinguish the activity performed from other activities not containing that object, thus leading to a rapid reduction of possibilities.

The following key caregiver requirements have provided the main guidelines for the design of our system:

- standardized task models: Applying standardized task models has many advantages. First of all, it means that operating experience already exists. In addition, for some of the task modeling languages, tools are available for design and simulation purposes.
- continuous monitoring: Another important requirement is continuous monitoring. Depending on the funding available, caregivers only see their clients a very short amount of time per day (in Germany, it is usually less than 10% of the time people are awake). During the rest of the day, people who might have officially been rated to be in need of help/support are left on their own. Continuous monitoring will support the process of identifying activities clients encounter difficulties with. In cases of severe cognitive deficit, clients might not even remember the incident correctly, or even at all, in order to report on it the next time they see their caregiver.
- prompt notification of behavior change: In order for the caregiver to be able to react in a timely manner to changes in behavior patterns, prompt notification is necessary. If the caregiver wants to address certain difficulties, it is important that the time period between their actual occurrence and the conversation about them is not too long. Otherwise, the client might not be able to discuss them anymore. Sometimes, it is even hard for a mentally fit person to remember what he/she had for lunch three days ago. Since certain activities only happen once a day, it should be sufficient if the system notifies the caregiver of potential behavior changes on a daily basis.
- intuitive system operation: The system should be intuitive to operate. A person who has never before worked with the system should be able to understand its output and know when to provide which input as soon as it becomes necessary.
- significant results: The results coming from the system should be significant. Systems that produce numerous false alarms are perceived to be annoying and worthless. Sooner or later, they will be deactivated (e.g., the Office Assistant Clippit). Therefore, it is our goal to develop a system that has a *F*_2_-measure of >95%.

### 3.1. Sensor Middleware

The sensor middleware must achieve two main goals: (i) physical and (ii) logical abstraction.

The main concern about physical abstraction is that the middleware must be able to operate with numerous hardware variants from different manufacturers. This is important because nobody would invest in a system that cannot guarantee that spare parts will be available for at least five years. Facilitating physical abstraction offers an easy way of switching from one manufacturer to the other. Various manufacturer-dependent drivers are dynamically loaded as required.

Logical abstraction is met by achieving transparency using a simple mapping mechanism. Raw sensor data is transformed into meaningful sensor events. The following context generation module does not rely on any specific hardware setup. It needs information about the meaning behind certain sensor events and not about the way they are connected to the infrastructure. We facilitate a very primitive yet powerful interface made up of simple labels (*i.e.*, strings). The labels are human readable. The sensor middleware could also be replaced by another piece of software as long as the same labels are issued on time. For example, instead of reporting “data acquisition module X has sensed a state change on channel Y”, the output might express, “toilet: presence detected”.

### 3.2. Context Generation

Based on prior output, the context generation module has to make sense of continuously incoming sequences of such events. By leveraging the common sense knowledge of caregivers, we have defined a set of ADLs utilizing the notation of task models. This set currently consists of the following nine activities: using the toilet, transferring, waking/sleeping, dressing, eating, washing, bathing, combing one's hair and napping. These nine activities are listed in order of significance. “Using the toilet” is said to be the most descriptive activity when it comes to assessing the degree of dependence an older person may encounter. The least significant activity for measuring this value (out of the nine mentioned) is “napping”.

**Figure 2.**
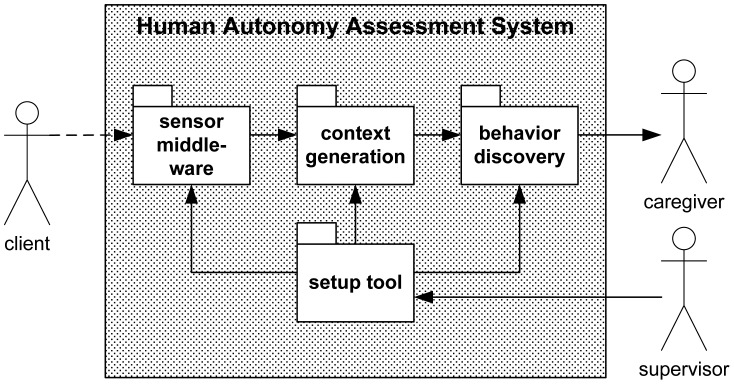
System process chain with four core modules (sensor middleware, context generation, behavior discovery and setup tool) and three roles (client, caregiver and supervisor).

With the help of the setup tool (see Figure 2), the supervisor can enable the appropriate ADLs. Depending on the physical condition of a client, it may happen that it does not make sense to monitor all possible ADLs. In order to save money, the supervisor might also decide to leave out certain ADLs so as to facilitate a less costly sensor installation. The “waking/sleeping” activity is mandatory since it is used to detect the beginning of a new day.

We use Paterno's Concur Task Tree (CTT) notation [31], because it has been shown to be quite intuitive to understand for both caregivers and computer scientists [32]. The reason for its intuitiveness lies in its compact and understandable representation. Nevertheless, it is very powerful and flexible when it comes to modeling temporal ordering and offers a rich set of operators [33]. Moreover, tools for design and simulation are publicly available (e.g., TERESA [34]).

Because CTT is a representational rather than an executable language, we had to transform the activity models into an executable equivalent. Since CTT is devoted to model concurrency and asynchronous behavior, we chose Petri nets (PNs) as the target language.

In the context of the formal modeling of computational processes, user interfaces and human-machine dialogue, PNs are of particular interest in a number of approaches. A short overview of the relevant literature can be found in [35].

Our transformation approach exploits the Theory of Regions [36]. The algorithm will search for typical structures (regions) within the CTT model and reproduce them in PN fashion. Up to now, the transformation supports the following temporal operators: sequential enabling, choice and independent concurrency. As for the remaining ones (i.e., concurrency with information exchange, deactivation, enabling with information passing, suspend-resume, iteration, finite iteration, optional tasks and recursion), so far we have not found an ADL for which they would be mandatory.

The reason we are using two formalisms is simple: In terms of intuitiveness, CTT outperforms PNs. On the other hand, PNs have major advantages when it comes to execution and validation. The caregiver will only interact with the CTT models of ADLs. PNs are generated automatically from the CTT models. The transformation process is straightforward. In order to demonstrate that the semantics of the CTT models are preserved, we would like to propose three transformation rules. The easiest in terms of transformation yet most common temporal operator within the ADLs is sequential enabling. For convenience, we use the same nomenclature as Paterno [31].

**Figure 3.**
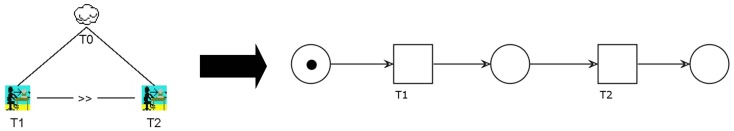
Transforming the sequential enabling-operator from CTT to PN representation.

The left side of Figure 3 illustrates two tasks, T1 and T2, which are subtasks of the abstract task T0. T1 and T2 are connected using the sequential enabling-operator, which can be written as T1 ≫ T2. This formula indicates that T1 has to be completed before T2 can start. In order to preserve this semantics, the PN on the right-hand side of Figure 3 inserts a simple place between T1 and T2. When T1 is completed, the token will move to the intermediate place. This guarantees that there is a point in time when T1 has finished before T2 starts. Afterwards, T2 can be executed. Finally, the token arrives at the place on the right.

**Figure 4.**
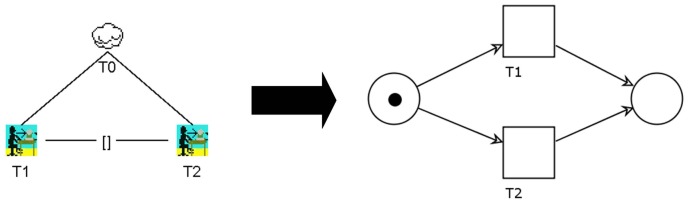
Transforming the choice-operator from CTT to PN representation.

The next transformation rule (see Figure 4) illustrates the choice between T1 and T2, which can also be denoted as T1 [] T2. The semantics behind this formula indicates that, as soon as a choice between T1 and T2 has been made, the other task can no longer be executed. The PN equivalent can be seen on the right side. Both tasks, T1 and T2, are connected by arcs starting at the initial place. The first task executed (*i.e.*, either T1 or T2) will remove the token from that place and move it to the final place. That way, only one task can be performed.

**Figure 5.**
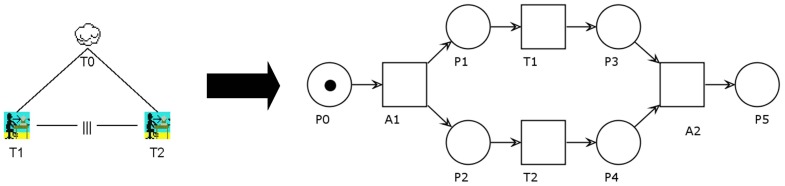
Transforming the independent concurrency-operator from CTT to PN representation.

The last transformation rule we would like to propose is shown in Figure 5. The independent concurrency operator allows tasks T1 and T2 to be performed in any order. They might also interleave each other. This means that the second may start before the first has finished. This behavior is in contrast to the sequential enabling described above.

The PN on the right side illustrates that this is the most complex transformation rule so far. In order to set up the concurrency, we have to introduce automatic transitions A1 and A2. A1 will initialize the interleaving by placing tokens in P1 and P2, respectively. From that point on, T1and T2 can be performed in any order, including nearly simultaneously. After all tasks have been completed, A2 will take care of synchronizing both paths. The final state of the PN shows only P5 marked. That way, it can easily be attached to further operators.

Each activity previously defined in a separate CTT will become a single PN. All PNs will run in parallel for the context detection. This will support concurrent occurrences of activities. The transitions of the PNs are triggered by the incoming events from the sensor middleware.

To implement the executable PNs, we chose TAPAAL [37], which supports multiple PNs within a single working space and is able to handle time constraints. Further information on TAPAAL can be found in [38].

The caregiver has to decide which activities are to be monitored for a specific client. The supervisor operates the setup tool called Editor. He/she will also define the start date of the monitoring process.

At the beginning of each day (detected using the dedicated “waking/sleeping” activity), every PN is set to its initial marking (state). As soon as a sensor event indicates that part of an ADL is taking place, the appropriate PN will experience progress.

Based initially on default values, but later using perceived knowledge, each activity (*i.e.*, its PN equivalent) has a dedicated timeout value. If the corresponding activity has not been completed by the time the timeout has been reached, the activity will be logged as “incomplete” together with information about its current state (*i.e.*, which events have already been perceived and which events were missing).

From a computational viewpoint, this approach is very resource-friendly since it is completely event-driven. Compared to activity recognition approaches based on labeled sensor data (e.g., Blanke and Schiele [39]), this is a fairly formal approach to context generation. From the caregiver's point of view, our rather strict methodology is comprehensible. Caregivers want to know exactly what their clients are able to do on their own and where they experience problems. Even if the person he/she is caring for is still responsible for himself/herself, the caregiver at least has a moral commitment and a social responsibility.

Besides the well-known deficiencies of HMMs when it comes to temporal relationships (Helaoui *et al.* [40]), purely probabilistic models will not support the recognition of wrong (*i.e.*, incomplete) ADL executions. Ideally, an incorrect execution of an ADL would lead to a reduced probability. Nevertheless, unless a threshold is defined, it is always possible to return the most probable activity currently being recognized.

The caregiver is not interested in what the current actions of his/her client are “kind of” like or which activity they most closely approximate; he/she wants to know whether certain (specifiable) criteria have been fulfilled or not.

### 3.3. Behavior Discovery

As illustrated by Figure 1, our system is based on a layered architecture. The behavior discovery tool takes the previously extracted information to the next layer, further compressing the information.

In the course of a day, every activity recognized by the context generation module (whether complete or incomplete) will be stored in an event log. The context generation and behavior discovery modules interface via an XML format for storing event logs called Mining extensible Markup Language (MXML) [41]. MXML [42] is a common data type in the data mining domain (*i.e.*, business process mining). Using a common meta-model allows for interoperability with other approaches (for both context generation and behavior discovery).

Process mining promises to extract new knowledge from the event log. In our case, the knowledge we are looking for is the typical daily routine (process structure) of a specific person. We evaluated several process-mining algorithms (Heuristics Miner, Fuzzy Miner, Alpha Miner and Transition System Miner) before coming to the conclusion that a configurable algorithm like the Transition System Miner (TSM) [43] best fits our needs: (i) to be able to check on process conformance and (ii) to be able to adjust the balance between under- and overfitting of the output.

TSM employs trace methodology A *trace* is a certain sequence of activities, while running TSM generates a transition system made up of states. These states describe parts of traces up to a certain point in time (or still to come, depending on the actual setting). TSM is configurable in five abstraction levels (maximal horizon, activity filter, number of activities, order consideration and visible activities). The possibilities arising from these abstraction levels can be seen in Section 5. TSM is able to produce a PN from the derived transition system.

Configuration of the mining algorithm is needed in order to achieve a balance between under- and overfitting of the resulting process model [43]. The algorithm applied to determine the metric will attempt to automatically find the best fit.

By utilizing the process conformance presented by [44], we can track whether current day observations already exist in the typical behavior seen so far. If so (*i.e.*, the sequence of activities for the current day conforms to the process model), the probability for that trace can immediately be determined by multiplying of the weights of all arcs on the path. If the opposite is true (*i.e.*, the course of the current day is atypical so far), the trace of the current day has to be merged with the typical behavior by reapplying TSM. Afterwards, it is also possible to compute its probability.

### 3.4. Towards a Realistic System Deployment

Since our approach is intended to be applicable even outside of the academic research field, it is also important to provide an initial estimation of the costs and deployment effort. The later mainly depends on the structure of the specific living area (*i.e.*, number of rooms in total, number of bathrooms, *etc.*). So far, we can say that on average it takes around 60 minutes per installation site.

Similarly the costs also depend on the structure of the specific living area and the amount of ADLs to be monitored. On average, two to three sensors are required for reliable detection. In total, nine ADLs have been defined. Since a certain infrastructure is always needed (worth roughly $350), it does not make sense to monitor fewer than three ADLs (typically waking/sleeping, going to the toilet and eating). With an average sensor price of approximately $80 (depending on the type of sensor), the estimated material costs are between $950 and $2,150 for the complete installation. All amounts mentioned refer to catalog prices.

Although this might seem very expensive at first glance, compared to the average costs insurance companies have to pay for institutional care, it corresponds roughly to one to three months depending on the care level needed. Another factor one should keep in mind is Moore's law: While the number of transistors doubles approximately every two years, the components containing them become cheaper and cheaper. This is especially true for the infrastructure costs, which are driven mainly by an embedded computer system, which executes the algorithms.

## 4. Metric for Anomalous Behavior Detection

Introducing the plain probability of each trace has three major disadvantages. First, the probabilities for days whose traces have different lengths (*i.e.*, the number of activities performed per day) are not comparable. This has to do with the way these probabilities are computed. Multiplying an additional probability (*p_i_* (x02208) (0,1]) can either result in the same value (if *p_i_* = 1) or reduce the final probability (if *P_i_* < 1). Second, the absolute value will decrease over time. This is due to the fact that the behavior model will grow (in terms of complexity) until every variation of the person's daily routine has been detected. Finally, it is not very meaningful for the caregiver.

Because of these disadvantages, we had to come up with a different measure (metric) for how typical a given day in the life of the client can be considered. Since context generation is based on checking ADL performance, this measure can also be understood as the perceived autonomy of the person under consideration. If the person “fails” in performing a particular activity, it will not be processed together with the other (completed) activities by the behavior discovery component. Therefore, the perceived behavior will differ from the typical behavior and lead to a reduced probability.

The metric *m* we want to propose for this purpose takes the plain probability mentioned above and scales it using the probability of the most probable path from the behavior model:

(1) $$m:=\frac{p_{M}(tr_{\text{current}})}{p_{M}(tr_{\text{highest}})}$$

In metric (1), *p_M_*(*tr*) denotes the probability of trace *tr* determined using the model *M*. While *tr_current_* represents the trace of the current day, *tr_highest_* corresponds to the trace with the highest probability. In addition to these definitions, we modified the well-known Dijkstra algorithm, which normally searches for the shortest path, to find the most probable path, thereby maximizing *p_M_*(*tr_highest_*).The modification is minimal: Instead of summing up the arc weights (costs), multiplication is used. The conditional check has to be adapted to search for the highest probability instead of the lowest costs.

Applying metric (1), we can rate how typical a certain daily schedule is compared to the most likely one ever perceived. Reconsidering the three disadvantages stated at the beginning of this section, we have so far overcome the second and third. Even if the structure of the model varies over time, scaling by the probability of the most probable path will guarantee that, after a while, there is no continuous reduction (because *tr_highest_* would also be influenced by that cause). To a caregiver, a scale from 100% (meaning the recently considered day is absolutely typical for the client) to *nearly* 0% (meaning a day like this has never been witnessed before) is very intuitive.

We have also come up with a mapping between this measure and the colors of a traffic light. Green would signalize a metric value of a “normal” day and red would stand for a relatively atypical day. Yellow would be used for anything in between (*i.e.*, near the dynamic threshold between red and green). This way we could fulfill the requirement calling for intuitiveness in terms of system usability. The next section will present an exemplary case.

## 5. Three-Day Example

To demonstrate the feasibility of our concept, we will now define three patterns representing three possible days. In addition to activity names, the event log also contains the three other elements of context: location, identity (*i.e.*, originator) and time (*i.e.*, timestamp). Additionally, the status of the denoted activity is stored (*i.e.*, complete or incomplete). Currently we are assuming a one-person household, so the originator field is a static value.

**Figure 6.**
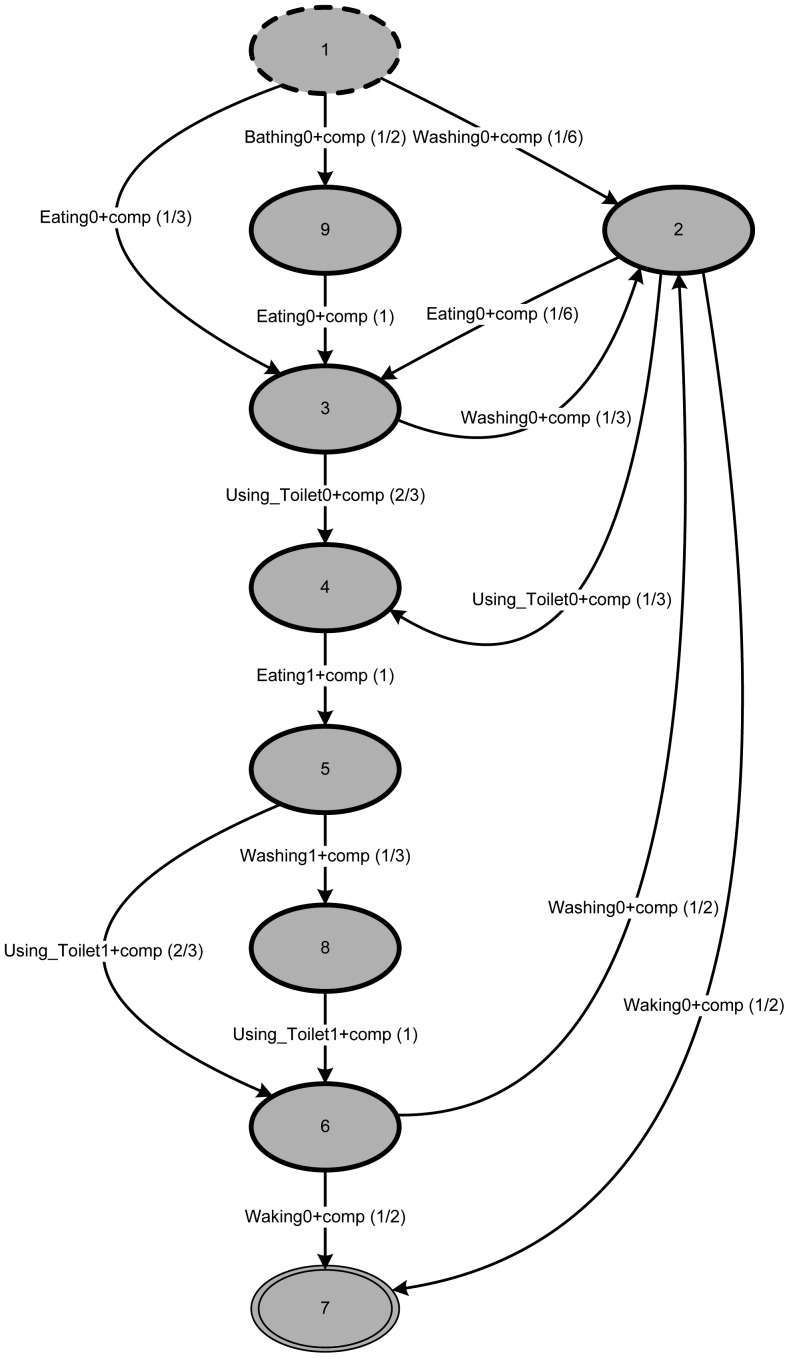
Typical behavior mined from the example event log in Table 1 using the Transition System Miner (TSM) with starting state 1 and final state 7; arcs labeled with activity names and probabilities (in parentheses).

Applying TSM with its default configuration (considering the five abstraction levels) to the event log produces a process structure representing the individual's behavior (based on the example event log in Table 1), like the one in Figure 6.

It is characteristic of this (default) representation that the model has *n* + 1 states (with *n* being the number of different activities within the event log). This is both the most compact and the most tolerant description achievable.

All three patterns from the event log are contained in the mined behavior. This can easily be seen by traversing the graph. In addition to the three patterns from the event log, other possible paths also exist. In general, this is a desirable feature of all models, including those acquired by process mining.

In the next step—trying to determine metric (1)—calculation *of p_M_*(*tr_highest_*) will use the path running from state 1 via state 2 directly to the final state, 7. This is certainly the shortest path (*i.e.*, most probable path using the arc probabilities) present in the graph, but has very little in common with any daily pattern. This can be observed by comparing the length of *tr_highest_* (*i.e.*,|*tr_highest_*| = 2) with the length of the traces found in the event log (*i.e.*, |*tr_highest_*| = 6 and 7). According to [43], the model presented in Figure 6 can be interpreted as underfitting (*i.e.*, the model allows for too many traces not present in the event log).

In order to determine a plausible metric, the mining algorithm has to be reapplied using a different configuration leading to a more balanced (in the sense of [43]) model. This time, we use the following configuration: *h* = 2, *F* = *A, m* = ∞, *q* = *sequence* and *V* = *A*. The maximal horizon *h* will limit the number of activities considered from the trace. Set *A* contains all possible activities; therefore, neither the setting for filter *F* nor the visible activities *V* will constrain the output. In other cases, the filter might be applied to place the focus on a subset of activities (e.g., considering only hygiene). The difference between the filter and visible activities is that activities being filtered out stay in the model but do not create separate states, whereas activities missing from the set of visible activities will be invisible in the resulting model.

The filtered result could be cut to *m* events, but with *m* = ∞ the original length is preserved. Using different settings for *q* (*i.e.*, bag or set) could remove the order of the trace, but, since we are interested in the temporal order of daily activities, *q* is left set to sequence.

Making use of the newly acquired model, reasonable values for *p_M_* (*tr_current_*) and *p_M_*(*tr_highest_*) can be determined. It turns out that metric (1) will result in $\frac{1}{3}$ for daily pattern *day*_0_, $\frac{1}{3}$ for *day*_1_ and 1 for *day*_2_. These results are exactly what one would expect, considering the event log in Table 1.

**Figure 7.**
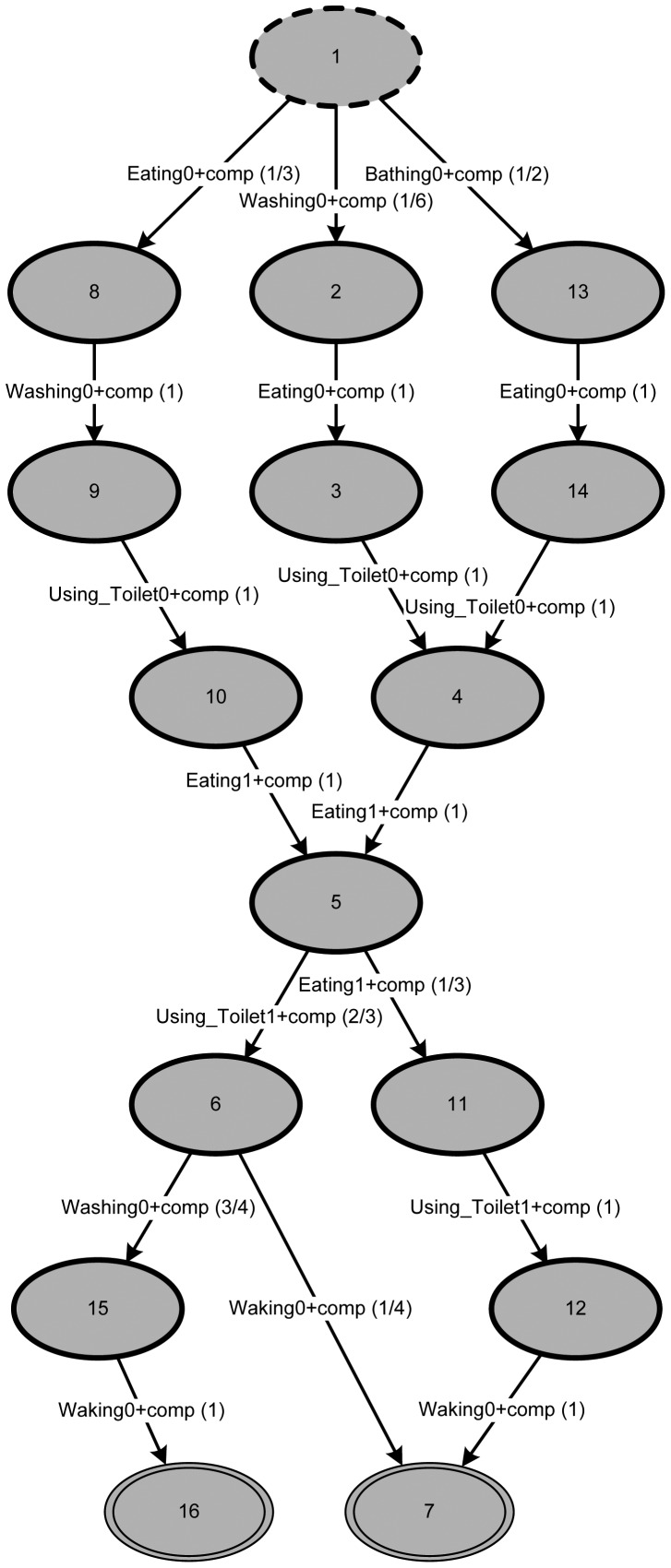
Typical behavior mined from the example event log in Table 1 using TSM (starting state 1 and final states 7 and 16); arcs labeled with activity names and probabilities (in parentheses).

In addition to the three daily patterns known from the event log, six extra ones can be seen in the model of Figure 7. Dividing the graph into two parts (the part above state 5 and the part below state 5) shows that in each case there are three possible paths: three paths from the starting state 1 to state 5 and three paths from state 5 to either final state (*i.e.*, state 7 or 16). Combing the permutations of both the upper and the lower parts of the graph will allow for nine different paths in total. Since three of them have already been introduced in the event log, the model has identified six additional ones.

Referring to the daily routine the graph represents, this suggests that the model is “prepared” to calculate behavior that has not been observed before. Let us assume the person the event log was produced by has three typical ways of starting the day in terms of ADLs (*i.e.*, the first half of the day, before lunch) and three ways of ending the day (*i.e.*, the second half of the day, after lunch). It seems very likely that those routines might mix one day resulting in a similar metric value.

In contrast, when a trace is observed that has not been observed before, in which case *p_M_*(*tr_current_*) will be particularly small, the metric will also provide very small values.

## 6. Evaluation

The evaluation section is divided into two parts: The first focuses on context generation while the second evaluates behavior discovery and the defined metric.

### 6.1. Functional Test of Context Generation with an Example

In order to prove the feasibility of the context generation module (*i.e.*, in terms of a proof-of-concept), we equipped a regular toilet with ambient sensors in our lab. We chose three simple binary sensors: one to detect a person sitting on the toilet, one to record toilet paper use and the last to sense the flushing of the toilet. For the first two indicators, we used Sharp's general-purpose distance-measuring sensor [45]. That infrared sensor type is very popular in the robotics community. The toilet flush indicator was a proximity sensor using Cypress' universal CapSense^®^ controller board [46].

**Figure 8.**
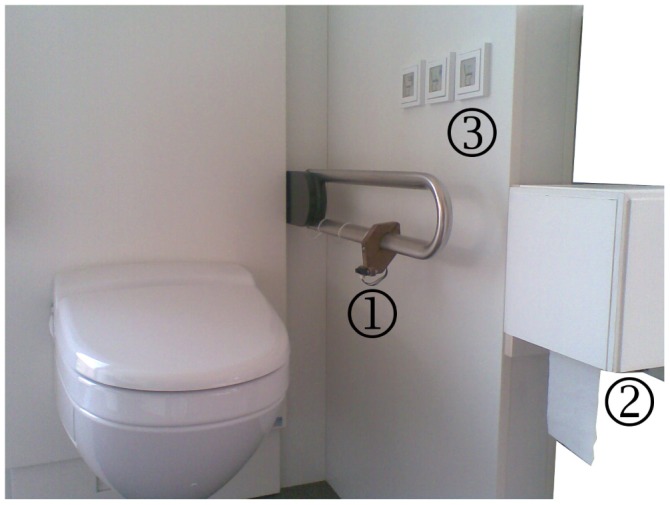
Toilet environment with three sensors: (1) toilet presence; (2) toilet paper use and (3) toilet flush.

The entire toilet environment, including the exact positioning of the above-mentioned sensors, can be seen in Figure 8.

After equipping the environment with hardware, the next step was to collect the data coming from those binary sensors. Inspired by the PROSAFE project [23], we used Advantech's data acquisition module [47] to do this. Data (*i.e.*, TCP/IP packets) coming from the acquisition modules is then processed by our sensor middleware using the appropriate driver. According to the current XML-based configuration, the middleware translates rather abstract sensor information into more expressive event-based knowledge (*i.e.*, a textual representation for every piece of sensor information).

Within the context generation, mapping occurs between those labels and the interaction task nodes of the CTT model of interest—in this case the “Using_Toilet” model. Actually, this mapping concerns the labels and the executable PN that was automatically generated by the CTT model, but, for reasons of simplicity and since both models are equivalent in terms of their behavior, a direct link between labels and CTT interaction tasks is assumed. This mapping describes the causal relationship between an event being observed by the sensors and its meaning with regard to the progress of ADLs specified by the CTT models.

**Figure 9.**
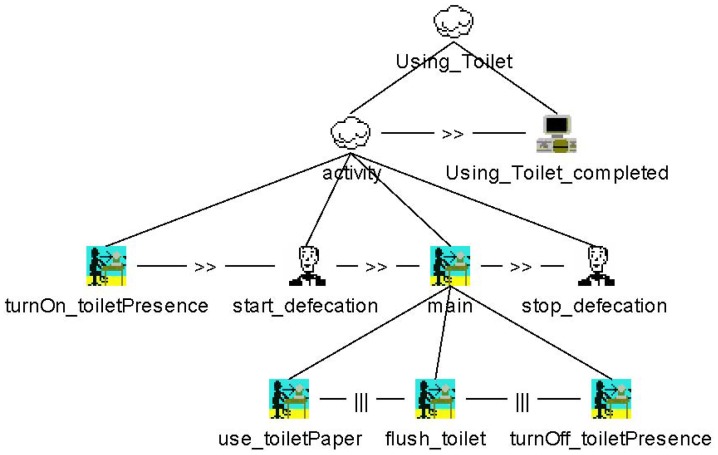
CTT model of the ADL “Using_Toilet” (including all six possible variations).

As an example, Figure 9 illustrates the possible steps of execution regarding the ADL “Using_Toilet”. The sequence is initiated when a person sits down on the toilet. After urinating or defecating, the person has three options: take a piece of toilet paper, flush the toilet or get up from the toilet. Once this decision is made, two possibilities remain. Finally, there is only one feasible way of ending the entire activity. Therefore, the number of possible permutations is calculated using the factorial operation.

When the entire process is finished and the model reaches the “Using_Toilet_completed” state (*i.e.*, the application task in CTT notation), the context generation module will automatically add the appropriate entry to the daily protocol. This entry contains all four elements of Dey's context: activity, identity, time and location.

In addition, the model tracks the duration of the entire process. The measurement starts as soon as the toilet is occupied and ends as soon as all three options of the “main” branch (*i.e., use_toiletPaper, flush-toilet* and *tumOff_toiletPresence*) have been completed. As can be seen in Figure 9, those points in time are expressed by the CTT user tasks *start_defecation* and *stop_defecation*. The following XML-code excerpt from the daily log sums up the whole entry:

<!— … —><Audit Trail Entry> <!— *Dey ‘s activity: Using_Toilet* + *complete* —> <Workflow Model Element>Using_Toilet</Workflow Model Element> <EventType>complete</EventType> <!— *Dey ‘s identity: Lisa* —> <Originator>Lisa</Originator> <!— *Dey ‘s time: December 7th 2011 @ 10:48 a.m.* —> <Timestamp>2011–12–07T 10:48:00.000 +01 :00</Timestamp> <Data>  <!— *Dey ‘s location: Bathroom* —>  <Attribute name=”Location”>Bathroom</Attribute>  <!— *duration of the defecation process in seconds* —>  <Attribute name=” defecation_duration_s”>103</Attribute> </Data></Audit Trail Entry><!— … —>

Experiments with ten students and colleagues aged between 25 and 35 from the inHaus Center have shown that the setup described above is capable of correctly detecting the specified activity. Of course, people of that age do not necessarily belong to the target group, but, for a proof-of-concept in general, people of any age can be used as test persons.

Events detected by the presence sensor as well as the flush sensor were very reliable (with an accuracy of nearly 100%). The only indicator showing a lower accuracy rate was the toilet paper usage sensor. This was due to the prototype design of the toilet paper dispenser. For the field test, a more reliable indication is advisable.

### 6.2. Realistic Simulated Evaluation of Behavior Discovery

To conduct an evaluation of behavior discovery, we will assume a fully functional context generation module. Carrying out the behavior discovery evaluation in a real world setting entails two main risks: (1) It is very time consuming and expensive in terms of equipment; and (2) there is no guarantee that the test persons will actually change their behavior during the study. There is no way of proving the overall system functionality if there is no variation in behavior.

Our approach is twofold: We collected actual sensor data coming from five individuals' living quarters and asked the caregivers to protocol the typical sequence of ADLs for each person over a one-week time period. We then enhanced the protocols by cross-checking their entries with the events emerging from the sensors.

For example, if the daily protocol noted that the test person got up at 8:30 a.m., we searched for related evidence within the sensor data collected. Since all rooms adjacent to the bedroom were additionally equipped with motion detectors (based on the EnOcean wireless standard [48]), we could easily retrieve the exact time. Similarly, if the caregiver's protocol suggested that the person normally took a nap after lunch, but the sensor data for a specific day indicated that he/she left the living area instead, we removed that entry from the protocol. This modified protocol was the source for our model of typical behavior.

Even the very first representations of these ADL behavior patterns showed that each person has a unique daily routine—like a “behavioral” fingerprint. While some people change their routines daily, others have a special routine for every single day of the week (*i.e.*, many parallel paths).

**Figure 10.**
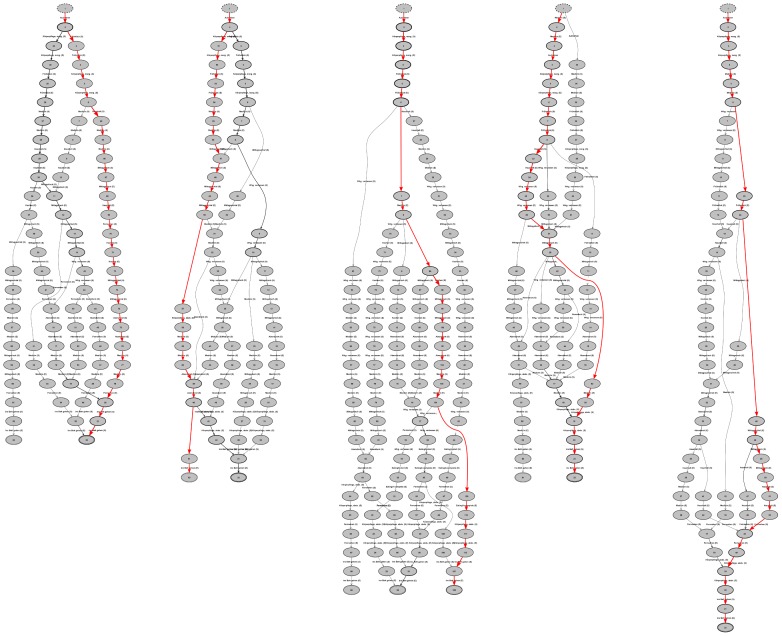
Typical daily behavior in terms of ADLs of five test persons P1–P5 (from left to right).

We also found indications that there is a difference between weekday (*i.e.*, from Monday to Friday) and weekend (*i.e.*, Saturday and Sunday) behavior. Figure 10 sums up our results in terms of typical behavior. The representation is equivalent to the one from Section 5 except that the path added/updated last is indicated in red. While the diagram is not intended to be readable in detail, it does show how diverse the ADL behavior of five different people can be.

Behavior discovery works on a daily basis. The reason for this was discussed in Section 3. Every day (*i.e.*, trace) in the typical behavior of any given individual starts with getting up in the morning and ends with going to bed.

Having generated the typical behavior for each person (*i.e.*, P1: female, 79 years; P2: female, 87 years; P3: male, 84 years; P4: male, 75 years and P5: male, 87 years), we were able to artificially (re-)produce further daily logs. This is due to the fact that the behavior models contain more variations than actually observed. The tool we utilized for this purpose was based on the work of Burattin and Sperduti [49].

From interviews with caregivers and the literature, e.g., [50], we know that there are four primary reasons for irregular behavior: (1) activities can be completely forgotten (removed); (2) they are delayed in time (delayed); (3) they are switched with other activities (swapped) and (4) they are performed more often than usual (repeated). The latter is the case if the person forgets that he/she has already completed a certain task (*i.e.*, retrospective memory error). This can be very dangerous if the task happens to be taking medicine.

Instead of waiting for P1–P5 to make mistakes in one of the four error classes, we artificially generated them starting with a “normal” trace. With this approach, we generated 70 days of data for each person. Every day consists of five versions: the normal one, a version with removed activities, a version with delayed activities, a version with swapped activities and a version with repeated activities. In total 350 traces were artificially generated.

The evaluation of the behavior discovery was intended to answer the following two questions:

- For how long does HAAS need to be installed and trained by the daily behavior of a person before it is able to distinguish between normal and abnormal days?
- What *precision* and *recall* can the caregiver expect when relying on HAAS?

**Figure 11.**
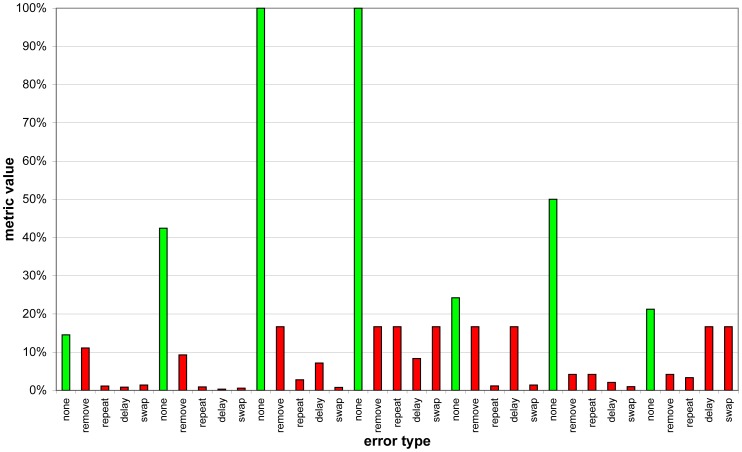
Metric values of P1 after 21 days (green: normal behavior; red: abnormal behavior).

Before we answer these questions, let us have a look at an example. Figure 11 shows the metric values of seven days in a row after the system has monitored P1 for three weeks. The green bar represents the “normal” version of the day, while the red bars denote “abnormal” days.

It can easily be seen that if we consider every day individually, the metric value of a normal day (green bar) is always greater than the metric value of every error type for that day (the following four red bars). Taking the whole week into account, at least for the example illustrated by Figure 11, it is impossible to find a threshold that satisfies a perfect classification. Choosing a metric value threshold of around 20% would lead to a *precision* of roughly 96.5% and a ratio of detected abnormal traces and totality of abnormal traces of 100%.

**Figure 12.**
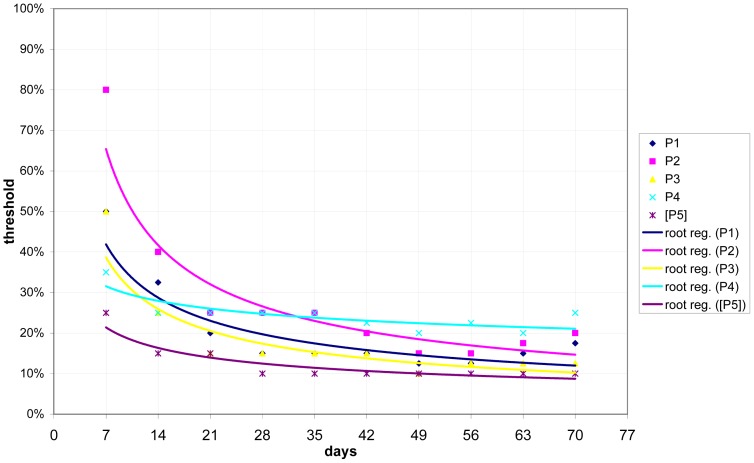
Dependency between optimal threshold and time (including root regression).

We determined that there is a relation between the time the system has been installed and trained and the optimal threshold. This dependency can best be described by a root function. Figure 12 illustrates this finding. The regression indicates that, during the first couple of days, a threshold of approximately 50% is most appropriate. After roughly six weeks, a threshold between 10% and 20% is more advantageous.

Utilizing these function characteristics as the dynamic threshold setting, we were able to answer the above-mentioned questions. Actually, both questions are closely related: *precision* and *recall* depend on the time the system is up. The longer the system is running, the higher the *precision* and *recall*. Similarly, it is impossible to answer how long the system has to be installed and trained without naming the *expected precision* and *recall* values. To combine both *precision* and *recall*, we chose the *F*_2_-measure.

P5's living quarters were not equipped with sensor hardware. Consequently, we were not able to cross-check the data from the daily protocol for that individual. That is why we disregard P5 in the following discussion. Additionally, we are aware of the fact that data for only four or five individuals is not statistically significant.

Table 2 presents the overall results regarding two different time periods. First, we calculated mean values and twofold standard deviation for the period running from two to ten weeks. Then, we compared those results to the second time period (six to ten weeks). Even after only two weeks, the mean *F_2_*-measure values are better than 96.87%. Taking the twofold standard deviation into account, this value is reduced to 90.5%.

Waiting another month slightly improves the mean values but drastically improves the twofold standard deviation. Now, for roughly 95% of all cases, the *F*_2_-measure values will be better than 98.07%, which we consider to be a quite promising result. For more details on the evaluation process, please refer to [51].

## 7. Conclusions and Future Work

We have presented a systematic approach to a novel human autonomy assessment system, abbreviated HAAS. With our metric, it is possible to rate how typical a person's daily behavior is when considering his/her activities of daily living (ADLs) in relation to his/her typical behavior.

Our approach differs greatly from recent approaches: Using an integrated formal approach to context generation, we are able to distinguish between complete and incomplete task executions. This enables caregivers to take a closer look at those tasks their clients have encountered difficulties with. The corresponding activities are defined in cooperation with the caregiver using task models. In comparison, classical activity recognition techniques using probabilistic models will provide only detection accuracy without further contextual information.

In our system, behavior discovery builds on the output of the context generation. After utilizing a standard exchange format, a process mining algorithm (*i.e.*, TSM) is applied. An automated balancing between under- and overfitting is performed. Finally, the presented metric can be determined.

We evaluated the context generation module using data gathered from our test bed, the CareLab located in the Fraunhofer inHaus Center in Duisburg, Germany. Additionally, we received data from real smart homes equipped with sensors. One problem we face is the granularity of sensor data originating from those facilities. In order to detect correct fulfillment of activities of daily living (or, at least, in order to do so with a high probability), a relatively fine sensor distribution granularity is required. This can certainly be realized in our laboratory environment, but is not necessarily available in normal households.

Using the dynamic threshold, we calculated that it is possible to map the metric value to the appropriate traffic light color. It had to be shown that all transitions are possible and that they occur at the right time. Performing this evaluation in a real-life situation would have been too time-consuming. Moreover, it could not be guaranteed that subjects would actually behave in an anomalous way during observation time. Therefore, we exploited expert evaluation. Utilizing real sensor data in combination with caregiver knowledge to produce simulated sequences, we were able to evaluate the traffic light signal in time lapse.

Since the system has proven its usefulness to caregivers, we will now further extend its functionality. Based on the typical behavior discovered, the system will also be able to cue the occupant as to which activities would typically come next (in case the occupant has suffered a blackout).

Although our work has concentrated on detecting ADLs and finding outliers over time, the system has a generic core. Depending on what kinds of tasks are specified (e.g., assembly line workflows), the system would also be able to identify irregularities in those tasks.

## Figures and Tables

**Table 1.** Event log containing three daily patterns.

Event log		
1× *day*_o_	2× *day*_1_	3× *day*_2_
Washing0+comp	Eating0+comp	Bathing0+comp
Eating0+comp	Washing0+comp	Eating0+comp
Using_Toilet0+comp	Using_Toilet0+comp	Using_Toilet0+comp
Eating1+comp	Eating1+comp	Eating1+comp
Using_Toilet1+comp	Washing1+comp	Using_Toilet1+comp
Waking0+comp	Using_Toilet1+comp	Washing0+comp
	Waking0+comp	Waking0+comp

**Table 2.** Mean *F*_2_-measure values for the denoted period applying the twofold standard deviation.

weeks	P1	P2	P3	P4	[P5]
2–10	**98.77**%	**96.87**%	**99.06**%	**98.65**%	**94.73**%
	+ 1.23%	+3.13%	+0.94%	+0.42%	+3.21%
	−2.78%	−6.37%	−1.76%	−0.42%	−3.21%
6-10	**99.77**%	**98.69**%	**99.71**%	**98.82**%	**95.78**%
	+0.23%	+0.62%	+0.29%	+**0**%	+2.32%
	−1.04%	−0.62%	−1.01%	−**0**%	−2.32%
